# *Bifidobacterium longum* Subspecies *infantis* Strain EVC001 Decreases Neonatal Murine Necrotizing Enterocolitis

**DOI:** 10.3390/nu14030495

**Published:** 2022-01-24

**Authors:** Shiloh R. Lueschow, Timothy J. Boly, Steven A. Frese, Giorgio Casaburi, Ryan D. Mitchell, Bethany M. Henrick, Steven J. McElroy

**Affiliations:** 1 Department of Microbiology and Immunology, University of Iowa, Iowa City, IA 52242, USA; shiloh-lueschow@uiowa.edu; 2 Department of Pediatrics, University of Iowa, Iowa City, IA 52242, USA; timothy-boly@uiowa.edu; 3 Department of Nutrition, University of Nevada, Reno, NV 89557, USA; sfrese@unr.edu; 4 Department of Bioinformatics, Metabiomics, Carlsbad, CA 92008, USA; gcasaburi@evolvebiosystems.com; 5 Evolve Biosystems, Inc., Davis, CA 95618, USA; rmitchell@evolvebiosystems.com (R.D.M.); bhenrick@evolvebiosystems.com (B.M.H.); 6 Department of Food Science and Technology, University of Nebraska, Lincoln, NE 68588, USA; 7 Department of Pediatrics, University of California Davis, Sacramento, CA 95817, USA

**Keywords:** Bifidobacteria, *B. infantis*, preterm birth, infant, newborn, necrotizing enterocolitis, probiotics, premature, cytokines, gastrointestinal microbiome

## Abstract

Necrotizing enterocolitis (NEC) is a disease mainly of preterm infants with a 30–50% mortality rate and long-term morbidities for survivors. Treatment strategies are limited and have not improved in decades, prompting research into prevention strategies, particularly with probiotics. Recent work with the probiotic *B. infantis* EVC001 suggests that this organism may generate a more appropriate microbiome for preterm infants who generally have inappropriate gut colonization and inflammation, both risk factors for NEC. Experimental NEC involving Paneth cell disruption in combination with bacterial dysbiosis or formula feeding was induced in P14-16 C57Bl/6 mice with or without gavaged *B. infantis*. Following completion of the model, serum, small intestinal tissue, the cecum, and colon were harvested to examine inflammatory cytokines, injury, and the microbiome, respectively. EVC001 treatment significantly decreased NEC in a bacterial dysbiosis dependent model, but this decrease was model-dependent. In the NEC model dependent on formula feeding, no difference in injury was observed, but trending to significant differences was observed in serum cytokines. EVC001 also improved wound closure at six and twelve hours compared to the sham control in intestinal epithelial monolayers. These findings suggest that *B. infantis* EVC001 can prevent experimental NEC through anti-inflammatory and epithelial barrier restoration properties.

## 1. Introduction

Preterm birth continues to represent a significant worldwide problem impacting one in every ten births, including 400,000 infants per year in the United States alone [1]. Premature birth results in the immaturity of almost all organ systems, and these infants are uniquely susceptible to a variety of diseases, including necrotizing enterocolitis (NEC) [2,3,4,5,6]. NEC is an inflammatory intestinal disease whose risk factors include prematurity (risk is inversely proportional to gestational age at birth), formula feeding, and microbiome dysbiosis (often characterized by a distinct spike in Enterobacteriaceae species shortly before NEC onset) [2,3,4,5,6]. Treatment strategies for NEC are limited; have not markedly changed in decades; have a variety of potential drawbacks; there are no current targeted therapies [7]. Thus, there is an increased interest in prevention strategies.

Intestinal dysbiosis is a risk factor for the development of NEC, and much attention has been given to the use of probiotics to prevent NEC [8,9,10]. Probiotics are live microorganisms that, when administered in adequate amounts, confer health benefits on the host [10,11]. The use of probiotics to prevent NEC has been a topic of study for several decades and was driven in part by the fact that historically, the only known preventative strategy for NEC is a human milk diet, whether mother’s own breast milk or donor breast milk [8,10,12,13,14,15,16]. While the mechanisms behind human milk’s protective effects remain unclear, it is thought to be due to a variety of factors, including intestinal maturation factors, passive immunity factors, and antimicrobial factors [2,12,13,14,15,16]. One possible mechanism behind these protective effects may be human milk oligosaccharides (HMOs) [16,17,18,19]. HMOs are the third most abundant macromolecule by mass in human milk but do not provide nutrition for infants as they are not digested by the infant [16,20,21]. Instead, they are hypothesized to serve a variety of functions in the gastrointestinal tract, but one of the most notable is as a prebiotic to guide the formation of an age-appropriate bacterial microbiota [16,17,18,19,20,21,22].

Probiotic use in preterm infants is one of the most robustly studied therapies in all neonatal medicine [8,9,10]. While many single and combination probiotic preparations have been shown to prevent NEC, *Bifidobacterium longum* subspecies *infantis* (*B. infantis*) is found in most common selections [10,23,24]. *B. infantis* has a unique, symbiotic relationship with humans because its genome contains up to five different HMO utilization clusters [23,24,25]. These gene clusters are devoted to the binding, transport, and breakdown of HMOs into components usable by the cell [23,24,25]. In doing so, *B. infantis* can completely consume HMOs inside the cell, thus preventing the utilization of HMOs or HMO-derived by-products by other competing bacteria, particularly pathogens [22,23,24,25,26]. Interestingly, the prevalence of *B. infantis* has declined in recent years, especially in developed countries, possibly a result of current neonatal intensive care unit practices such as the prevalence of antibiotic usage, cesarean section delivery, formula feeding, and sanitary improvements [22,25,27].

Despite both a strong biological rationale and literature supporting the use of *B. infantis* or other probiotic strains to prevent NEC, there remain significant questions in the field regarding both the use and safety of probiotics. While probiotic use has been studied in over 50,000 infants in 29 countries, there are concerns about the quality and heterogeneity of the studies, including the strains used, dosage, excipient carrier (milk, formula, water), timing of administration, and the duration of administration [28,29]. In addition, in the US, most probiotics are classified by the FDA as a food supplement, more specifically as a food for special dietary use or medical food, instead of a drug [29,30]. This classification provides less regulation and ultimately may contribute to wide strain variation and quality in the available products [28,29]. Lastly, probiotic-induced sepsis of preterm infants has been reported in the literature, raising questions about safety [28]. These concerns led to a recent statement from the American Academy of Pediatrics (AAP) urging caution in the use of probiotics in extremely vulnerable premature neonates, noting weak scientific evidence supporting probiotics lead to a decrease in rates of NEC and late-onset sepsis [29].

A recent probiotic product has been developed, which may not only serve as a more natural colonizer of the infant’s gut but also has the potential to confer health benefits such as decreasing susceptibility to diseases such as NEC. *B. infantis* EVC001 is a strain of *B. infantis* that possesses all five HMO utilization clusters and has been found to positively modulate the microbiome and reduce enteric inflammation in preterm infants [30,31,32,33,34,35,36]. This probiotic strain is monitored to produce consistent bacterial composition and abundance in each dose and to prevent contamination [31,32]. However, to date, studies using *B. infantis* EVC001 have not been published in animal models or clinical trials for the prevention of NEC. To begin to address this gap in the knowledge, we utilized our well described murine models of NEC in combination with EVC001 with and without a carrier HMO to understand its ability to prevent experimental NEC.

## 2. Materials and Methods

### 2.1. Animals

C57Bl/6J mice were acquired from Jackson Laboratories (Bar Harbor, ME, USA) and then bred and raised at the University of Iowa according to protocols approved by the Institutional Animal Care and Use Committee (Protocol #8041401). Postnatal day (P)14–16 mice were used in our experiments as they are intestinally equivalent, in terms of gene regulation, to a 22- to 24-week human infant [37]. Neonates were housed with their mothers in the vivarium and dam fed until the day of the experiment. On the day of the experiment, mice were separated from their mothers and were maintained in a temperature- and humidity-controlled chamber. Mice were randomly chosen from each litter and assigned to each treatment group to prevent litter bias. Additionally, mice were sexed, and male and female mice were balanced within each group.

### 2.2. EVC001 Dose Optimization

**Figure 1:**
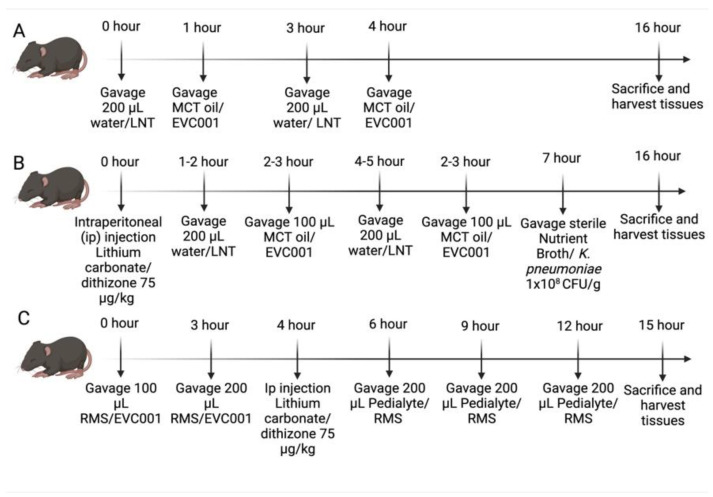
Model schematic. (**A**) Timeline for dosing optimization trials with Lacto-N-Tetraose (LNT) and EVC001. (**B**) Timeline for the Paneth cell disruption with bacterial dysbiosis NEC model as well as the incorporation of the dosing scheme for LNT and EVC001. (**C**) Timeline for the RMS NEC model with the incorporation of EVC001 dosing. RMS, Rodent Milk Substitute.

P14-16 C57Bl/6J mice were initially given a 200 µL gastric gavage of the HMO Lacto-N-Tetraose (LNT) (Jennewein Biotechnologie GmbH, Rheinbreitbach, Germany) at a concentration of 0.015 g/mL or water as a vehicle control (Figure 1A). One hour later, 125 µL, 175 µL, or 250 µL MCT oil was given as an oral gavage (Figure 1A). This cycle was repeated three hours later (Figure 1A). To keep hydrated during the model, pups were given oral gavages of 200 µL of Pedialyte (Abbott labs, Columbus, OH, USA) approximately every 6 h. Sixteen hours after the first oral gavage of LNT/water, serum was collected via facial vein puncture to analyze serum cytokines and then mice were sacrificed. Tissues were harvested, including serum for cytokine quantification, ileal tissue for injury scoring, and the cecum to perform microbiome analysis. Mice given EVC001 followed the same timeline but were given an oral gavage in volumes of 25 µL, 50 µL, or 100 µL in place of MCT oil at intervals matching the one-hour and four-hour time points shown in Figure 1A. For further rationale, please refer to Section 3.1.

### 2.3. NEC Models

Our experiments utilized the Paneth cell disruption model of NEC as we have previously described [38,39]. In brief, mice were given an intraperitoneal injection of 75 µg/kg of dithizone (Sigma-Aldrich, St. Louis, MO, USA) dissolved in 25 mM lithium carbonate solution followed six hours later by a gastric gavage of 1 × 10^8^ colony forming units (CFU)/g body weight of *Klebsiella pneumoniae* (*K. pneumoniae*) 10,031 (ATCC, Manassas, VA, USA) (Figure 1B). *K. pneumoniae,* before gastric gavage, was grown overnight and then subcultured and allowed to grow to mid to late log phase in Nutrient Broth (NB) (BD Biosciences, Sparks, MD, USA) at 37 °C while being shaken at 200 RPM. Sham controls (*n* = 6), utilized equivalent volumes of lithium carbonate and sterile NB (BD Biosciences, Sparks, MD, USA), were used as vehicle controls. The dithizone (Dith) control group (*n* = 9) received dithizone as described above, sterile NB, water, and MCT oil. The *K. pneumoniae* (Kleb) control group (*n* = 3) received lithium carbonate, *K pneumoniae* as described above, water, and MCT oil. The LNT control group (*n* = 8) received lithium carbonate, sterile NB, LNT, and MCT oil. The *B. infantis* EVC001 (EVC001) control group (*n* = 4) received lithium carbonate, sterile NB, water, and *B. infantis* EVC001. Finally, the LNT plus *B. infantis* EVC001 (LNT+EVC001) control group (*n* = 4) received lithium carbonate, sterile NB, LNT, and *B. infantis* EVC001. NEC mice (*n* = 10), HNEC mice (*n* = 10), EvNEC mice (*n* = 10), and EvHNEC mice (*n* = 10) all experienced NEC induction as described above. Mice in the probiotic or probiotic control groups were given 200 µL of the HMO, LNT (Jennewein Biotechnologie GmbH, Rheinbreitbach, Germany)/water and 100 µL MCT oil/EVC001 as seen in Figure 1B. *Bifidobacterium longum* subspecies *infantis* (*B.* infantis) was provided by Evolve Biosystems (Davis, CA, USA) at a concentration of 8 billion CFU/0.5 mL already prepared in MCT oil suspension and kept at −20 °C until ready to utilize [36]. Upon beginning the experiment, the *B. infantis* EVC001 suspension was dethawed, mixed, and given via oral gavage. MCT oil was similarly already prepared and provided by Evolve Biosystems (Davis, CA, USA). Mice were monitored for the duration of the experiment and given Pedialyte (Abbott labs, Columbus, OH, USA) to maintain hydration at six-hour intervals as needed. At sixteen hours after dithizone delivery, serum was harvested via facial vein puncture from the mice to quantify inflammatory cytokines. Then mice were sacrificed, and the ileal small intestinal tissue, the cecum, and contents of the colon were harvested to look at injury scoring and the microbiome, respectively.

To remove the potential confounding impact of *K. pneumoniae* use in the Paneth cell disruption model, we also employed our recently described RMS formula-Paneth cell model [40]. In brief, P14-16 mice were given an intraperitoneal injection of 75 µg/kg of dithizone (Sigma-Aldrich, St. Louis, MO, USA) dissolved in 25 mM lithium carbonate or an equivalent volume of lithium carbonate alone as a vehicle control. One hour prior to injection with dithizone/lithium carbonate, mice were given an initial gavage of 200 µL of Rodent Milk Substitute (RMS) formula or Pedialyte (Abbott labs, Columbus, OH, USA) as a control [41]. The RMS formula or Pedialyte gavage was then given every three hours for a total of four feeds (Figure 1C). *B. infantis* EVC001 was centrifuged at 5000 RPM for 5 min, and the MCT oil was removed. *B. infantis* EVC001 was resuspended in an equivalent volume (one milliliter) of RMS formula and was given to mice in a total of two doses exactly as was performed in the Paneth cell disruption with bacterial dysbiosis NEC model. *B. infantis* EVC001 was then given three hours before the initial gavage of RMS with an equivalent volume of the RMS formula given as a vehicle control (Figure 1C). Mice in the probiotic group were given an oral gavage of 100 µL EVC001 in RMS formula or 100 µL RMS formula for the first dose, as seen in Figure 1C. For the second dose, EVC001 was diluted at a 1:1 ratio in RMS formula so that 200 µL of RMS could be given while still receiving the same amount of probiotic. Sham mice (*n* = 5) received Pedialyte (Abbott labs, Columbus, OH, USA) instead of RMS formula, lithium carbonate instead of dithizone, and RMS instead of EVC001. Dithizone (Dith) control mice (*n* = 5) received Pedialyte (Abbott labs, Columbus, Ohio, USA), dithizone, and RMS instead of EVC001. RMS control mice (*n* = 5) received RMS, lithium carbonate, and RMS instead of EVC001. EVC001 control mice (*n* = 6) received RMS, lithium carbonate, and EVC001. RMS NEC mice (*n* = 7) Ev RMS NEC mice (*n* = 7) experienced NEC as described above. In addition, as above, mice were monitored for the duration of the experiment, and at the 15-hour time point, serum was collected via facial vein puncture for analysis of serum cytokines and then mice were sacrificed (Figure 1C). Following euthanasia, ileal intestinal tissue was harvested to perform injury scoring, and then the cecum and colon contents were collected to measure the microbiome.

### 2.4. Injury Scoring

Ileal intestinal injury scoring was performed by a single blinded investigator as previously described [38,39,40,42,43]. In brief, the distal 1/3 of the intestine just before the cecum was harvested and then fixed in 10% buffered formalin, embedded in paraffin, and then sectioned at five µm thickness. The samples were stained with Hematoxylin and Eosin (H&E) and then evaluated under a Nikon microscope. NEC injury was scored on a five-point scale, with zero being healthy intestine to four being full-thickness necrosis. Scores were given based on the degree of degradation of villus architecture as well as lift from the basement membrane. Scores at or above two are considered significant and consistent with NEC.

### 2.5. Serum Cytokine Measurements

Blood obtained from the facial vein puncture was placed on ice for one hour following collection and then centrifuged at 7000 RPM for five minutes to isolate the serum [38,39,40]. Cytokines were quantified using a Meso-Scale Discovery V-Plex Assay (Meso-scale, Gaithersburg, MD, USA) according to the manufacturer’s instructions, with the plates being read on a Sector Imager 2400 at 620 nm. All samples were run in duplicate.

### 2.6. Microbiome Analysis

DNA was extracted from the cecum and colonic contents (fecal) by first bead beating using a FASTPREP-96™ high-throughput bead beating grinder and lysis system (MP Biomedicals, Irvine, CA, USA) for 5 min at 1800 rpm. Lysates were then processed using the ZymoBIOMICS 96 Magbead DNA Kit (Zymo Research, Irvine, CA, USA), adapted for the ThermoFisher Scientific Kingfisher Flex platform (Waltham, MA, USA) and then quantified using the QuantIT dsDNA kit, High Sensitivity (Waltham, MA, USA). Then, 16S rDNA sequencing of the V4 domain was performed as described in Frese et al., 2017 [31], using the Earth Microbiome Project standard protocols (www.earthmicrobiome.org, accessed on 20 October 2021) [44]. PCR amplicons were grouped at roughly equivalent amplification intensity ratios and then purified using the NucleoSpin Gel and PCR Clean-up (Macherey-Nagel, Dueren, Germany). The PCR amplicons were then submitted to the UC Davis Genome Center DNA Technologies Core for Illumina paired-end library preparation, cluster generation, and 2 × 300 base pair paired-end Illumina MiSeq run at the UC Davis Genome Center. The resulting data from the sequencing run was analyzed using the QIIME software package (University of Colorado, Boulder, CO, USA, version 2 2021.8.0) [45]. Sequences were demultiplexed, and then DADA2 was used as a quality filter with default settings to exclude chimeric and low-quality sequences while splitting sequences into 100% operational taxonomic units (OTUs)/amplicon sequence variants (ASVs) with respective frequencies [46]. DADA2 was also used to truncate the dose tailoring microbiome data at the 300 nucleotide mark; the Paneth cell disruption with bacterial dysbiosis NEC model data at the 240 nucleotide mark; and the Paneth cell disruption with RMS formula NEC model data at the 250 nucleotide mark in response to the observation of a reduction in sequence quality in the reverse reads beyond those thresholds [46]. A secondary filtration was completed to remove low-abundance ASVs at a threshold of 0.005%. Then filtered ASVs were classified taxonomically using the Greengenes 16S rRNA database (gg_13_8 release) [47]. Relative abundances of various taxa were gleaned from a combination of the ASVs classification as well as the frequency counts of the various ASVs from the DADA2 step. Furthermore, EMPeror weighted UniFrac principal coordinate analyses (PCoAs) were generated to examine beta diversity [48].

### 2.7. Quantitative PCR

Quantification of the total *B. infantis* was performed by quantitative real-time PCR using Blon_2348 sialidase gene primers Inf2348F (5’-ATA CAG CAG AAC CTT GGC CT-3’), Inf2348_R (5’-GCG ATC ACA TGG ACG AGA AC-3’), and Inf2348_P (5’-/56-FAM/TTT CAC GGA/ZEN/TCA CCG GAC CAT ACG/3lABkFQ/-3’) [49]. Each reaction contained 10 μL of 2× TaqMan Universal Master Mix II with UNG master mix (ThermoFisher Scientific, Waltham, MA), 0.9 µM of each primer, 0.25 µM probe, and 5 μL of template DNA. Thermal cycling was performed on a QuantStudio 3 Real-Time PCR System and consisted of an initial UNG activation step of 2 min at 50 °C followed by a 10-min denaturation at 95 °C, succeeded by 40 cycles of 15 s at 95 °C and 1 min at 60 °C. Standard curves for absolute quantification of CFUs were generated using genomic DNA extracted from a pure culture of *B. infantis* EVC001 with a known concentration [36].

### 2.8. Wound Healing Assay

Rat ileal epithelial cells (IEC-18, ATCC, Manassas, VA, USA) were maintained in Dulbecco’s modified Eagle’s medium (DMEM) with 4 mM L-glutamine, 10% fetal bovine serum, 100 U/L penicillin G, 100 U/L streptomycin C, and 0.05 U/L insulin. Prior to experimental conditions, cells were plated on 35 mm cell plates and grown to 95% confluence. Cells were then starved in DMEM with 0.5% fetal bovine serum, with 100 U/L penicillin G and 100 U/L streptomycin C. Culture medium supernatant from *B. infantis* EVC001 grown in RPMI 1640 medium (Thermofisher, Waltham, MA, USA) plus 2% glucose for 16 hours (Evolve BioSystems, Davis, CA, USA) was then added at 1 and 10% dilutions. Cells were starved for 48 h. Following the starvation period, 3 circular wounds were made on each plate using a drill press with a modified silicone bit [50]. Following wounding, the cells were again starved for 48 h, without reapplication of the EVC001 supernatant. After the second starvation period, the positive control cells were treated with mouse recombinant epidermal growth factor (mrEGF) 10 ng/mL. Untreated cells were used as a negative control. Each wound was then observed by microscopy (Nikon Eclipse Ts2, NIS Elements Version 5.20.02, 40×) at 0, 6, 12, 24, and 48 h. Sham (*n* = 3), EGF (*n* = 4), RPMI-10 (*n* = 11), EVC001-1 (*n* = 17), and EVC001-10 (*n* = 13) all reached the 70% or greater wound healing cut-off at the 48 h time point to be included in the analysis. At each time point, the area of each wound and percent closure from time 0 were calculated.

### 2.9. Statistics

Murine experiments were performed using animals from at least two separate litters to reduce potential litter bias. Wound healing assay experiments were performed at least in triplicate for each group, and all wounds that at least healed to the 70% threshold by the 48-h time point were included in the analysis. Sample sizes are denoted in the figure legends and are explicitly listed within the methods. One- or two-way ANOVAs were performed using a Dunnet’s or Tukey’s multiple comparisons test within Graph Pad Prism (version 9.2.0, San Diego, CA, USA) when the data was determined to be normally distributed. When data was determined to be non-normally distributed, non-parametric Kruskal–Wallis tests were performed within Graph Pad Prism version 9.2.0. Significance was defined as *p* values ≤ 0.05. In graphs where a summary data point is shown, the data point is considered to be the average. All error bars shown in the figures are reported as the standard error of the mean (SEM).

## 3. Results

### 3.1. EVC001 Delivery to Mice at Human Relevant Concentrations Causes Intestinal Injury and Inflammation Due to MCT Oil Toxicity

**Figure 2:**
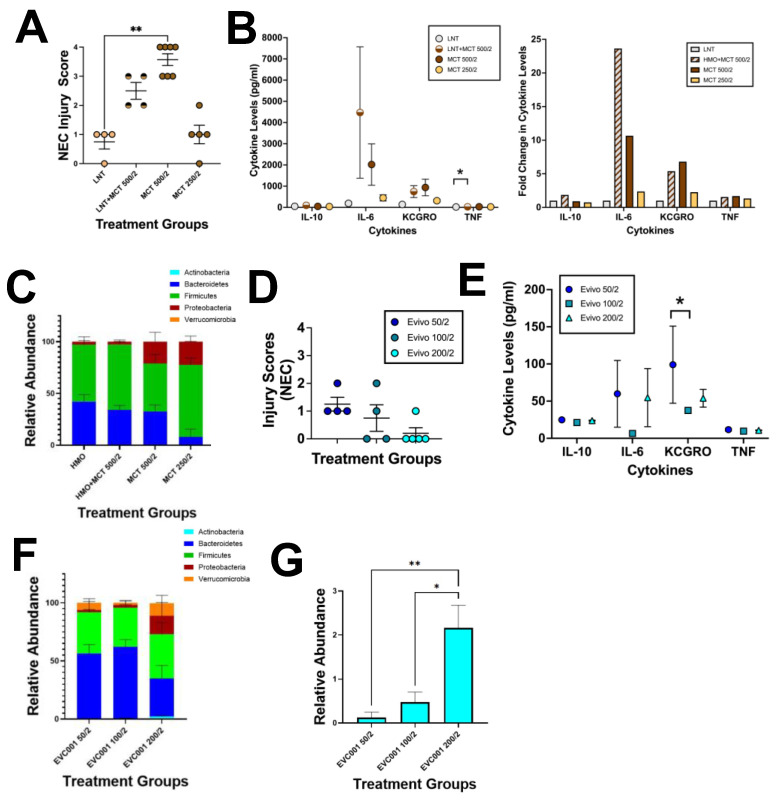
MCT oil in human-relevant doses is toxic to mice and must be tailored to size. (**A**) NEC injury scores for mice receiving MCT oil with or without gavage of LNT (*n* ≥ 4 mice per group). (**B**) Serum cytokine analysis for IL-10, IL-6, KC-GRO, and TNF for mice given MCT oil with or without gavage of LNT (*n* ≥ 4 mice per group). (**C**) Cecal microbiome analysis of the phylum-level relative abundances for mice given MCT oil with or without gavage of LNT (*n* ≥ 4 mice per group). (**D**) NEC injury scores for mice receiving varying doses of probiotic EVC001 (*n* ≥ 4 mice per group). (**E**) Serum cytokine analysis for mice receiving varying doses of EVC001 (*n* ≥ 4 mice per group). (**F**) Cecal microbiome analysis of the phylum-level relative abundances for mice given varying doses of EVC001 (*n* ≥ 4 mice per group). (**G**) Cecal microbiome Bifidobacteriaceae family level relative abundances for mice given varying doses of EVC001 (*n* ≥ 4 mice per group). For all *p* values, * ≤ 0.05, ** ≤ 0.01.

Probiotics are well described in the literature to prevent experimental NEC in murine models [8,9,10]. Interestingly, in almost every published study, the animal model is given an equivalent dosage of probiotics to what their human counterparts would receive even though neonatal mice weigh 5–10 g compared to very low birth weight (VLBW) human preterm infants that weigh from 500–1500 g. *B. infantis* EVC001 is also somewhat unique in the probiotic field as it is suspended in MCT oil instead of being lyophilized and later suspended in breast milk or dextrose containing fluids for feeding via a nasogastric or orogastric tube. Thus, we first wanted to determine if MCT oil would be tolerated by P14-16 mice, especially given their small size. In human infants, the EVC001 probiotic is given as a 500 µL dose, but P14-16 mice are not able to safely tolerate oral gavages greater than 250 µL. Thus, multiple smaller volumes were given to achieve a total 500 µL daily dose. Given the ultimate intention was to test EVC001 along with gavages LNT, LNT diluted with water was also given to the mice. LNT was selected as a source HMO as it would be a more relevant substance to serve as sham than saline but was likely to act similar to saline gavages and give NEC injury scores of zero [38,39,40]. Additionally, EVC001 can utilize LNT as a sole carbon source, but LNT on its own has previously been shown to not affect experimental NEC scoring [35,51]. P14-16 mice are not able to safely tolerate oral gavages greater than 250 µL, so multiple smaller volumes were given to achieve a total 500 µL daily dose. Mice that received two doses of 250 µL of MCT oil to equal a daily dose of 500 µL had significant intestinal injury (greater than or equal to two), which was also significantly higher than the LNT alone group when MCT oil was gavaged without LNT (*p* ≤ 0.0025), but not with LNT (*p* = 0.1964) (Figure 2A). In contrast, mice that received 125 µL of MCT oil per dose had injury scores that were not statistically different from LNT levels and were also below the threshold of significance (less than 2) (Figure 2A). In addition to histologic injury, we also examined the effect of MCT oil on serum cytokine levels to inform the ideal dosing scheme. Mice that received doses of MCT oil that reached a total of 500 µL had trending but non-significant increases in IL-10, IL-6, and KC-GRO compared to mice that received HMO alone (Figure 2B). Additionally, mice that received 500 µL total of MCT oil had significant increases in TNF-α compared to their counterparts only receiving LNT (*p* ≤ 0.0382) (Figure 2B). In contrast, mice that received a total of 250 µL of MCT oil had inflammatory cytokine levels akin to the HMO levels (Figure 2B). When looking at the microbiome of these mice, it was observed that doses of MCT oil without HMO gavage resulted in trending higher levels of Proteobacteria with compensatory decreases in Firmicutes (MCT 500/2) or Bacteroidetes (MCT 250/2) (Figure 2C). To verify that addition of EVC001 would not alter our findings, mice were given two separate doses of EVC001 suspended in MCT oil with total end volumes of 50 µL, 100 µL, and 200 µL. These three dosing regimens had similar average injury scores (Figure 2D) and cytokine levels (Figure 2E), which were comparable to levels seen with LNT alone (Figure 2A, B). Interestingly, along with the trending but non-significant increased injury observed in EVC001 50/2, there was a significant increase in KCGRO between EVC001 50/2 and EVC001 100/2 (*p* ≤ 0.0194). Additionally, the microbiome of these three groups was similar except for a trending increase in Verrucomicrobia in the mice that received 200 µL total volume of EVC001 that resulted in a compensatory decrease in Bacteroidetes levels (Figure 2F). It was notable that although the relative abundance of Bifidobacteriaceae was very low in the three groups, the relative abundance in the mice that received 200 µL total volume (2 gavages of 100 µL) of EVC001 had levels that were significantly higher than the other two groups (*p* ≤ 0.0236) (Figure 2G). This confirmed that normal preterm infant doses of EVC001 were harmful to P14-16 mice due to the MCT oil carrier, but the lower, more tailored dose of 200 µL total was well tolerated.

### 3.2. EVC001 Supplementation Decreases NEC Injury without LNT Supplementation

**Figure 3:**
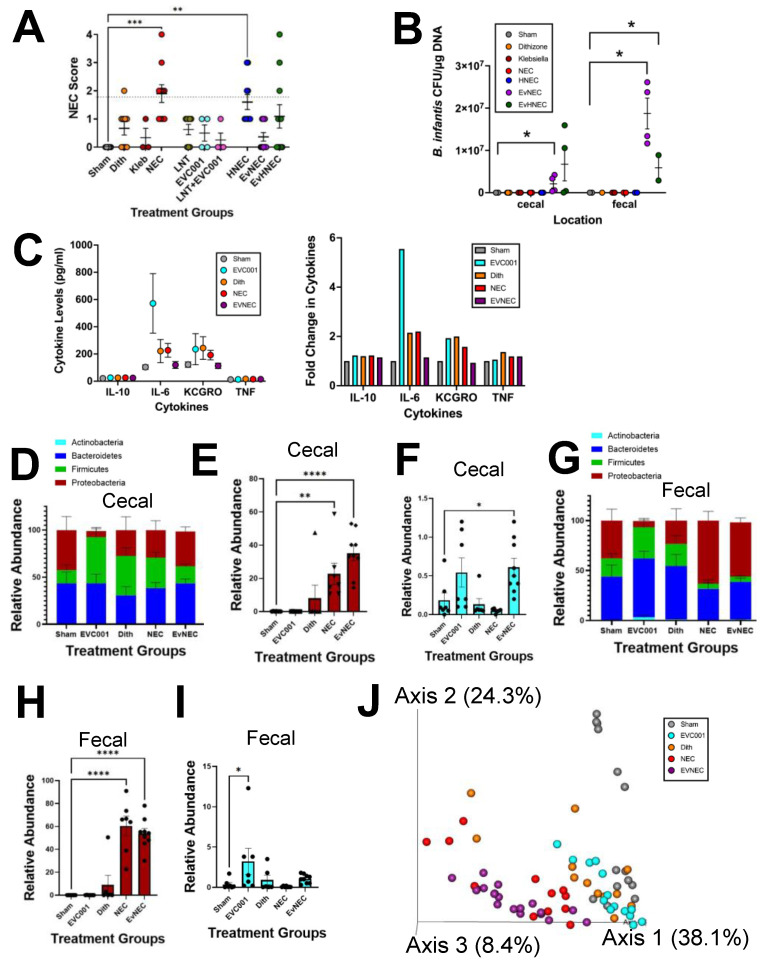
EVC001 decreases experimental NEC injury scores in the Paneth cell disruption with bacterial dysbiosis model. (**A**) NEC injury scores for control mice, NEC mice, and NEC mice receiving LNT (HNEC), without LNT, but with EVC001 (EvNEC) or with LNT and EVC001 (EvHNEC) (*n* ≥ 3 mice per group). (**B**) Quantitative PCR probing for *B. infantis* within the cecal and fecal microbiome (*n* ≥ 3 mice per group). (**C**) Serum cytokine levels for IL-10, IL-6, KC-GRO, and TNF and the representative fold changes. (**D**) Phylum level relative abundances for the cecal microbiome (*n* ≥ 6 mice per group). (**E**) Enterobacteriaceae family level relative abundances for the cecal microbiome (*n* ≥ 6 mice per group). (**F**) Bifidobacteriaceae family level relative abundances for the cecal microbiome (n ≥ 6 mice per group). (**G**) Phylum level relative abundances for the fecal microbiome (colon contents) (*n* ≥ 6 mice per group). (**H**) Enterobacteriaceae family level relative abundances for the fecal microbiome (*n* ≥ 6 mice per group). (**I**) Bifidobacteriaceae family level relative abundances for the fecal microbiome (*n* ≥ 6 mice per group). (**J**) Weighted UniFrac principal coordinate analysis plot including cecal and fecal samples within each group (*n* ≥ 6 mice per group). For all *p* values, * ≤ 0.05, ** ≤ 0.01, *** ≤ 0.001, and **** ≤ 0.0001.

We next evaluated the effect of EVC001 on our Paneth cell disruption with the bacterial dysbiosis NEC model (Figure 3A). Mice in the LNT, EVC001, and LNT+EVC001 groups all had non-significant injury scores that were similar to sham controls (*p* ≥ 0.7225), and mice with NEC induction resulted in statistically higher injury compared to sham controls (*p* ≤ 0.0003). The addition of the HMO LNT had no impact on NEC injury scores (HNEC group versus NEC *p* > 0.9999), and the injury scores were still significantly higher than sham control (*p* ≤ 0.0015). In contrast NEC animals that received EVC001 (EvNEC versus NEC *p* ≤ 0.0019; EvHNEC versus NEC *p* = 0.1577) had injury scores that were similar to sham controls (EvNEC versus sham *p* > 0.9999; EvHNEC versus sham *p* = 0.1697). Interestingly, although gavage of EVC001 alone resulted in a significant decrease in injury scores compared to NEC and scores not statistically different from sham, gavage of both EVC001 and LNT had injury scores not statistically different from NEC or the sham group (Figure 3A). These results suggested that LNT was not important for EVC001 to impact injury scores as originally anticipated and, in fact, had the opposite effect of mitigating the effect of EVC001 to a small degree. To determine if EVC001 (B. infantis) was truly present in the small and large intestine of the mice after gavage, subspecies-specific qPCR was performed to quantify EVC001. As expected, only animals that received EVC001 had the presence of *B. infantis* EVC001 as measured by qPCR in the cecum as well as colon contents (Fecal) (Figure 3B). It was notable that in the cecal compartment, NEC mice that received *B. infantis* EVC001 had significantly increased levels of *B. infantis* DNA present compared to sham (*p* ≤ 0.0472), but the levels were not significantly different between EvNEC and EvHNEC (*p* > 0.9999) (Figure 3B). Similarly, in the fecal compartment, mice that received *B. infantis* EVC001 had significantly increased levels of *B. infantis* DNA present compared to sham (*p* ≤ 0.0364), but there was no significant difference between EvNEC and EvHNEC (*p* > 0.9999) (Figure 3B). As the addition of LNT did not make a significant difference in injury score or the capacity of *B. infantis* to colonize in the small or large intestine, studies using LNT were excluded from further analysis. When analyzing cytokine levels, no significant differences were observed in IL-10, IL-6, KC-GRO, or TNF between sham animals and NEC animals regardless of gavage of *B. infantis* EVC001 (Figure 3C). Finally, the microbiome was analyzed and compared (Figure 3D–I). In the cecal microbiome, while Proteobacteria levels were equivalent between sham animals and all NEC animals, Enterobacteriaceae levels, of which K. pneumoniae is a member, were significantly higher in NEC animals (*p* ≤ 0.0001) and EVC001 + NEC (EvNEC) animals (*p* ≤ 0.0001) compared to sham controls (Figure 3D,E). It was also notable that in contrast to what was seen in the qPCR data (Figure 3B), the cecal microbiome Bifidobacteriaceae levels were relatively low in animals that received *B. infantis* EVC001 (Figure 3F). Although the *B. infantis* alone group did not have statistically higher relative levels of Bifidobacteriaceae compared to sham, the animals that received *B. infantis* in the NEC group did (*p* ≤ 0.05) (Figure 3F). Similarly, in the fecal microbiome at the phylum level, sham control animals had equivalent levels of all phyla compared to all NEC animals regardless of probiotic administration, except for Proteobacteria, where NEC animals had statistically higher levels compared to sham animals (*p* ≤ 0.0093) (Figure 3G). When looking more in-depth at Enterobacteriaceae, all NEC animals, regardless of probiotic administration, had significantly higher levels compared to sham controls (*p* ≤ 0.0001) (Figure 3H). Again, although contrasting with the stark difference observed in the qPCR data, EVC001 gavage resulted in relatively low levels of Bifidobacteriaceae overall (Figure 2I). Although *B. infantis* alone mice had significantly higher levels compared to shams (*p* ≤ 0.05), NEC mice receiving *B. infantis* EVC001 had levels equivalent to sham in contrast to the opposite trend displayed in the cecal microbiome (Figure 3F,I). Finally, when examining the beta diversity through a weighted UniFrac PCoA, it was observed that NEC mice, regardless of the delivery of probiotic EVC001, clustered together (red and purple circles), but distinctly from control mice (gray, orange, and aqua circles) (Figure 3J). These results taken together suggest that EVC001 delivery was able to significantly decrease NEC injury without the presence of LNT and did so with minimal impact on cytokine levels or the relative composition of the microbiome.

### 3.3. Effects of EVC001 Are Model Dependent

**Figure 4:**
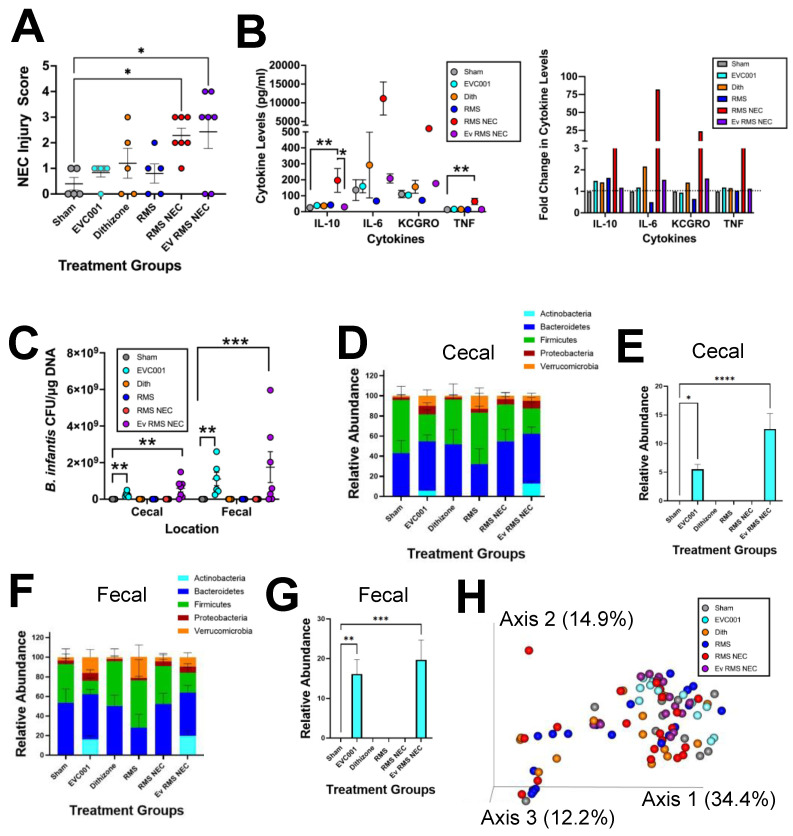
EVC001 efficacy in decreasing experimental NEC injury is model dependent. (**A**) NEC injury scores for control mice, RMS formula NEC mice, and RMS formula NEC mice receiving EVC001 (Ev RMS NEC) (*n* ≥ 5 mice per group) (**B**) Serum cytokine levels for IL-10, IL-6, KC-GRO, and TNF and the representative fold changes (**C**) Quantitative PCR probing for *B. infantis* EVC001 DNA within the cecal and fecal microbiome (*n* ≥ 5 mice per group) (**D**) Phylum level relative abundances for the cecal microbiome (*n* ≥ 5 mice per group) (**E**) Bifidobacteriaceae family level relative abundances for the cecal microbiome (*n* ≥ 5 mice per group) (**F**) Phylum level relative abundances for the fecal microbiome (colon contents) (*n* ≥ 5 mice per group) (**G**) Bifidobacteriaceae family level relative abundances for the fecal microbiome (*n* ≥ 5 mice per group) (**H**) Weighted UniFrac principal coordinate analysis plot including cecal and fecal samples within each group (*n* ≥ 5 mice per group). For all *p* values, * ≤ 0.05, ** * ≤ 0.01, *** ≤ 0.001, and **** ≤ 0.0001.

One drawback of utilizing the Paneth cell disruption model of NEC is that it is dependent on *K. pneumoniae* gavage to induce dysbiosis. This may confound probiotic studies through bacterial interactions. To compensate for this, we also utilized a complimentary RMS formula NEC model, which induces NEC through dithizone-induced disruption of Paneth cells and RMS formula feeding [40]. An additional advantage of this model is that it exhibits higher serum cytokine levels compared to the standard Paneth cell disruption with bacterial dysbiosis NEC model [40]. While EVC001 significantly improved NEC injury in the Paneth cell disruption with the bacterial dysbiosis model, EVC001 did not impact injury in the RMS model (Figure 4A). RMS formula NEC mice and RMS formula NEC mice receiving EVC001 (Ev RMS NEC) had significantly higher injury scores compared to sham controls (*p* ≤ 0.0201, 0.0358, respectively) (Figure 4A). Interestingly, when the cytokine levels were analyzed, RMS formula NEC mice had significantly higher levels of IL-10 compared to sham (*p* ≤ 0.0017), which was mitigated by gavage of EVC001 in the Ev RMS NEC group (*p* ≤ 0.0228) (Figure 4B). Similarly, for TNF-α, RMS formula NEC mice had significantly elevated levels compared to sham (*p* ≤ 0.0075) (Figure 4B). Although Ev RMS NEC mice had TNF-α levels not significantly different from sham (*p* > 0.9999), the levels were also not significantly different from RMS NEC (*p* = 0.2411). Furthermore, although not significantly different, it was notable that levels of IL-6 and KC-GRO had trending decreases in RMS formula NEC mice given gavage of EVC001 compared to their NEC counterparts (Figure 4B). Similarly, to the Paneth cell disruption with bacterial dysbiosis model results, upon examining the total abundance of *B. infantis* EVC001 via qPCR in the cecum and colonic contents (fecal), it was clear that gavage of *B. infantis* EVC001 (aqua and purple circles) resulted in elevated levels of EVC001 DNA detected, while groups not receiving *B. infantis* EVC001 (gray, orange, blue, and red circles) did not display this trend (Figure 3B and Figure 4C). In addition, similar to the results from the Paneth cell disruption with bacterial dysbiosis model, mice that received EVC001 had significantly increased *B. infantis* EVC001 DNA present in the cecal and fecal compartments compared to sham (*p* ≤ 0.0073 and *p* ≤ 0.0025 respectively) (Figure 3B and Figure 4C). RMS formula NEC mice that received *B. infantis* EVC001 also had significantly higher amounts of *B. infantis* DNA compared to sham controls in both the cecal and fecal compartments (*p* ≤ 0.0009 and *p* ≤ 0.0017, respectively) (Figure 3B and Figure 4C). When looking at the microbiome of these mice, there were no significant differences in any phyla between NEC and NEC+EVC001 treatments (Figure 4D,F). In contrast to observations in the Paneth cell disruption with bacterial dysbiosis NEC model, the RMS formula NEC model did not have a high relative abundance of Proteobacteria in any group (Figure 4D,F). Additionally, mice that received EVC001 in the RMS formula NEC model saw significantly larger amounts of Bifidobacteriaceae present in both the cecal and fecal microbiome compared to all other treatment groups (*p* ≤ 0.0083) and larger relative abundances than what was observed in the Paneth cell disruption with bacterial dysbiosis NEC model (Figure 3F,I and Figure 4E,G). Mice that received EVC001 without NEC induction also had significant increases in Bifidobacteriaceae levels compared to shams in both the cecal and fecal compartment (*p* ≤ 0.0437). These results carried over into a beta diversity analysis using a weighted UniFrac PCoA, as mice that were given EVC001 either as a control (aqua circles) or in the NEC condition (purple circles) tended to cluster together in the plot although not distinctly from the other groups displayed (Figure 4H).

### 3.4. EVC001 Improves Intestinal Epithelial Wound Healing

**Figure 5:**
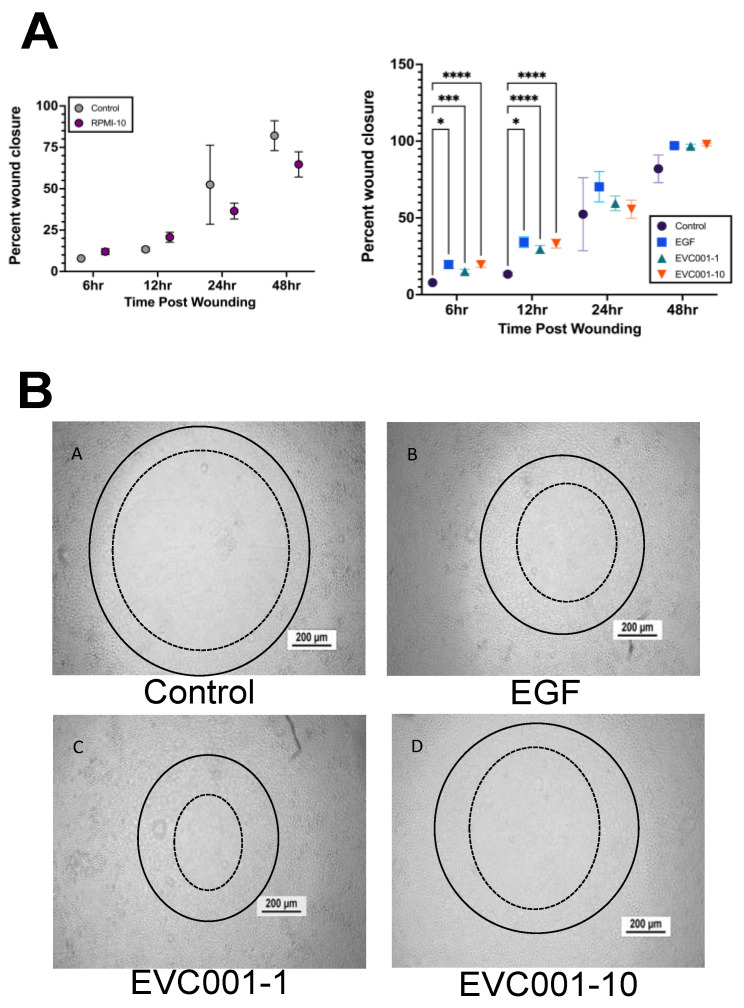
Wound healing assay results show *B. infantis* EVC001 metabolites can improve intestinal epithelial cell wound healing. (**A**) Compared wound healing results between sham and RPMI and results from the wound healing assay (*n* ≥ 3 wounds per group) (**B**) Representative microscopy images of the epithelial cells with the original wound size at the 0-h time point (solid line) added for comparison to the degree of healing at the 12-h time point (dashed line). For all *p* values, * ≤ 0.05, *** ≤ 0.001, and **** ≤ 0.0001.

Recent studies have provided support for *B. infantis* having a benefit in protecting and healing the epithelial barrier of the intestine [10,34,36,52]. To determine whether this could be a potential mechanism for the decrease in injury scores observed in mice receiving EVC001, radial wounds were made in rat intestinal epithelial cell monolayers (IEC-18) 48 h following treatment with EVC001 and wound closure was compared over 48 h to sham controls and wounds treated with EGF (positive control) (Figure 5). RPMI media, which served as the vehicle control for the EVC001 supernatants, did not show any significant difference in wound closure compared to shams at any time point examined (6 h and 12 h *p* = 0.1339, 24 h *p* = 0.5753, 48 h *p* = 0.3520) (Figure 5A). At both the 6- and 12-hour time points after wounding, both concentrations of EVC001 supernatants induced modest but significantly improved wound healing compared to the sham controls (*p* ≤ 0.0004) (Figure 5). Representative images from the 12-h time point after wounding are shown in Figure 5B, which were generated by making a solid line circle on the 0-h image and putting it onto the 12-h time point image and adding a dashed line indicative of where the wound had healed to at that point (the inner edge of cellular migration and proliferation).

## 4. Discussion

NEC remains the leading cause of intestinal morbidity and mortality in preterm infants and induces long term complications such as short bowel syndrome and neurodevelopmental delays for survivors [2,3,4,5,6]. Unfortunately, treatment strategies have been limited and non-targeted, which has driven research into prevention strategies such as the use of probiotics to manipulate the microbiome towards a more healthy and stable microbial community [8,9,10]. However, despite significant research into probiotics, there remains no consensus on the use of probiotics, and the AAP has recently released a statement cautioning against the use of probiotics noting limited sound scientific evidence as to the benefits [29]. One of the more promising probiotics in the neonatal field is *B. infantis* due to its role as a substantial member of the healthy microbiome of infants; for its ability to consume HMOs; for its potential to stimulate wound healing and intestinal epithelial cell health; the potential for reduction in enteric inflammation; and the significant impact on immune cell polarization in vitro [8,9,10,23,24,25,35,52,53,54]. EVC001 is a recently developed *B. infantis* probiotic strain that was selected for its ability to completely digest HMOs and its ability to positively modulate the microbiome and reduce enteric inflammation [31,32,33,34,35,36]. However, to date, EVC001 has not been studied in animal models or clinical trials for the prevention of NEC. To begin to address this gap in knowledge, we utilized our well described murine models of NEC in combination with EVC001 with and without a carrier HMO to understand its ability to prevent experimental NEC. Our novel data shows crucial evidence that EVC001 significantly decreases experimental NEC and reduces inflammatory serum cytokine levels. Our data also show that these effects are model-dependent and act independently of significant modulation of the pathogenic Enterobacteriaceae members of the microbiome. We show that EVC001 exposure significantly increases wound healing of intestinal epithelial cells. We also, for the first time, show the importance of tailoring the dosing of probiotics in animal models, especially for those that use MCT oil as a vector. This is important as recommended servings for preterm infants are as much as 1000 times larger on a per-kg basis compared to P14-16 mice. Taken together, our novel data suggest that *B. infantis* strain EVC001 can decrease intestinal injury due to NEC potentially through decreases in inflammatory cytokines, stable colonization of the microbiome, and improvements in intestinal wound healing, making it a potential candidate for human trials in premature infants at risk for NEC.

Our results support that EVC001 decreases experimental NEC using the Paneth cell disruption and bacterial dysbiosis NEC model (Figure 3A). This is supported by other studies that have shown the beneficial properties of other *B. infantis* strains against NEC [8,9,10]. Interestingly, EVC001 was unable to significantly impact NEC rates in our RMS formula NEC model (Figure 4A). One possible explanation for this is the difference in delivery method. The *B. infantis* EVC001 in RMS formula NEC model was treated differently than standard practice and was subjected to centrifugation and resuspension in RMS formula rather than the standard MCT oil. This may have resulted in an overall decrease in viable cells, contrary to the results shown in the qPCR and microbiome data, which depict DNA presence, but not the viability of the cells (Figure 3B,D–I and Figure 4C–G). This could also potentially be due to the different mechanisms of action the two models work through. One potential mechanism that distinguishes the two models is their level of inflammation [40]. While both complementary models are inflammatory, the RMS formula NEC model induces much higher levels of serum cytokines relative to the Paneth cell disruption with bacterial dysbiosis model at similar time points (Figure 3C and Figure 4B) [38,39,40]. Strikingly, the Paneth disruption with bacterial dysbiosis NEC model showed uncharacteristically low levels of inflammation in the NEC mice overall, which could be due to collecting the serum at a later time point than previously published works after the cytokine storm had subsided [38,39]. To this point, EVC001 induced a significant decrease in serum IL-10 and a trending decrease in TNFα and IL-6 in the RMS formula NEC model (Figure 4B) but had no effect in the Paneth cell disruption with bacterial dysbiosis model (Figure 3C). Our RMS formula NEC model agrees with data published in a different formula-induced rat model of NEC, which showed significant decreases in the cytokines IL-6, IL-8, TNFα, and iNOS when *B. infantis* was given compared to the NEC mice not receiving the probiotic [8,55]. Therefore, it is possible that EVC001 is either working directly to decrease the inflammatory properties of formula, or the Paneth cell disruption and bacterial dysbiosis induced inflammation was not significant at the time point we examined to see effects.

Another suggested mechanism by which *B. infantis* can improve intestinal health is through decreasing intestinal epithelial cell permeability and enhancing tight junctions in intestinal epithelial cells [10,34,36,52]. Adding to this, our data shows improved wound healing when conditioned medium from *B. infantis* EVC001 was put onto wounded epithelial cells compared to the sham control (Figure 5). This would agree with improvements in barrier integrity and offers a further potential mechanism of action for the decrease in NEC injury in mice given *B. infantis* compared to those that did not.

Our results showed that the HMO, LNT, had no significant impact on NEC injury scores on its own (Figure 3A), which supports previously published work depicting the importance of additional sialyl groups necessary for efficacy [51]. The results seen with LNT contrast other studies using HMOs such as 2′FL [56,57] and DSLNT [51], which have been shown to be able to decrease NEC injury scores in rodent models as well as in human infants [51,56,57,58]. HMOs work through various mechanisms. While 2′FL is believed to regulate toll-like receptor 4 as well as eNOS levels and ultimately regulate the degree of inflammatory signaling and the amount of blood flow to the intestine, respectively [56,57], DSLNT is thought to work through glycan-binding receptors such as galectins and sialic-acid-binding immunoglobulin-like lectins due to the conformational necessity of the two sialyl groups and not having a fucosyl group [51]. It has also been hypothesized that HMOs function as prebiotics to aid in the establishment of a healthier microbiome [16,17,18,19,20,21,22]. We chose to use LNT for our studies as it has been noted that *B. infantis* EVC001 can utilize LNT as a sole carbon source [35]. Interestingly, we found that LNT did not impact quantities of *B. infantis* (Figure 3B and Figure 4C) or reduce NEC (Figure 3A). It is possible that the timing of delivery of LNT in our model compared to when *B. infantis* was given did not allow for the two to physically interact. This could be further supported by the fact that Bifidobacteriaceae levels were much higher in the RMS formula NEC model compared to the standard bacterial dysbiosis NEC model (Figure 3F,I and Figure 4D,F). In the RMS formula NEC model, it is possible that there are components of the RMS formula that the *B. infantis* preferred to utilize rather than colonize, leading to a higher relative abundance.

Lastly, our data is one of the first to examine the dose response effects of probiotics [8,10]. Traditionally, animal models of NEC have utilized 1 × 10^8^ CFU dosing per day, which is also the standard daily dosage given to preterm human infants even though they are 100–1000 times larger. To put the comparison into context, the standard dose of *B. infantis* EVC001 in humans is 8 × 10^9^ CFU per dose in a volume of 0.5 mL. Most probiotic preparations are modeled in term and late preterm infants with an average weight of around 2500 g. In contrast, the average weight of P14-P16 mice utilized in this study is around five to seven grams. This means the human infant is roughly 500 times the size of the mice used in our models. When considering dose per gram, human infants are receiving roughly 3.2 × 10^6^ CFU/gram, while the same dosing scheme is equivalent to 1.6 × 10^9^ CFU/gram in mouse pups. Even after reducing the dosing scheme to 40%, the mice still received greater than the 1 × 10^8^ CFU traditional probiotic dosing utilized in other models with different probiotics. Our data was born of necessity as we found that MCT oil in doses considered safe for preterm infants produced dose=dependent intestinal injury in neonatal mice, forcing us to examine smaller, more size-appropriate servings of EVC001 [59]. A typical preterm infant is given no more than 3 g of fat/kg/day. MCT oil is used as no more than 30% of the daily fat intake in preterm infants, or 0.9 g/kg/day. MCT oil contains 0.93 g fat per ml. Our mice that experienced MCT oil-induced injury received two daily doses of 0.250 mL MCT oil for a total dose of 0.5 mL or 0.47 g of fat per day but only weighed 5 g. This means that they received 94 g/kg/day of fat or roughly 100 times what is given to human preterm infants and explains why MCT oil was harmful. Importantly, our data show that probiotics were still effective at a much smaller dose. While this may seem trivial at first, it has very important real-world implications in the probiotic-NEC literature. The clinical trial literature for probiotics has shown mixed results of efficacy [8,10]. While this has been assumed to be because of trial heterogeneity, it may also be due to some element of bacterial drift. In the recent Probiotics in Very Preterm Infants (PiPS) trial in the UK, there was no difference in NEC rates in infants given *Bifidobacterium breve* compared to those receiving placebo [10,58]. However, the authors noted that many infants in the placebo arm of the study had intestinal colonization by *B. breve* [10,60]. Although in Figure 3B, it was notable that in our study there was no drift of *B. infantis* EVC001 into the other groups, this was likely due to being housed in separate isolates, in separate locations, and in a more controlled environment, which is not feasible in a clinical trial. As our dose of roughly 4 × 10^7^ *B. infantis* was just as effective as other published work using the standard 1 × 10^8^ *B. infantis*, it is possible that some probiotic studies with bacterial drift to placebo controls picked up enough colonization to offer some protection and cloud clinical trial interpretation.

## 5. Conclusions

NEC remains one of the most devastating diseases of the preterm infant. Despite decades of study, we are still searching for better treatments and preventative measures. While probiotic use offers potential, the American Academy of Pediatrics (AAP) recently urged caution in the use of probiotics in extremely vulnerable premature neonates, as there is a paucity of sound scientific evidence showing probiotics lead to a decrease in rates of NEC and late-onset sepsis. Thus, this data comes at an important time as it suggests *B. infantis* EVC001 may be a potential candidate for NICUs to utilize in premature neonates to decrease the incidence of NEC as well as the long-term complications that come with this devastating disease. Additionally, our novel data begin to elucidate how *B. infantis* EVC001 is able to reduce experimental NEC. Using our murine models, *B. infantis* EVC001 altered NEC-induced inflammation and increased wound healing. We anticipate this data will lead to clinical trials and further studies of EVC001′s mode of action.

## Data Availability

The data presented in this study are available on request from the corresponding author.
